# The Future of Computational Linguistics: On Beyond Alchemy

**DOI:** 10.3389/frai.2021.625341

**Published:** 2021-04-19

**Authors:** Kenneth Church, Mark Liberman

**Affiliations:** ^1^ Baidu Research, Sunnyvale, CA, United States; ^2^ University of Pennsylvania, Philadelphia, PA, United States

**Keywords:** empiricism, rationalism, deep nets, logic, probability, connectionism, computational linguistics, alchemy

## Abstract

Over the decades, fashions in Computational Linguistics have changed again and again, with major shifts in motivations, methods and applications. When digital computers first appeared, linguistic analysis adopted the new methods of information theory, which accorded well with the ideas that dominated psychology and philosophy. Then came formal language theory and the idea of AI as applied logic, in sync with the development of cognitive science. That was followed by a revival of 1950s-style empiricism—AI as applied statistics—which in turn was followed by the age of deep nets. There are signs that the climate is changing again, and we offer some thoughts about paths forward, especially for younger researchers who will soon be the leaders.

## 1 Introduction: Better Together

We are going to speculate about the future of Computational Linguistics (CL)—how things may change, how we think things should change, and our view of the forces that will determine what actually happens. Given that different people have different views of what the field is, and even what it should be called, we will define the field of Computational Linguistics by what is discussed in top venues, using Google Scholar’s ranking of venues.^1^ The name of one of these venues, the Association for Computational Linguistics (ACL), was controversial in the 1960s. The current name dates back to 1968.^2^ Before that, the name of the society included the phrase, “Machine Translation,” a topic that was more popular before the ALPAC report (Pierce and Carroll, 1966) than after the ALPAC report, especially among funding agencies in America, for reasons described by Hutchins (2003) among others. Since then, the field has changed directions a number of times for a number of reasons, as will be discussed below. Given this history of change, it is likely that there will be more changes in the future. One of the reviewers asked us to define the field in a way that will stand up to the test of time, but unfortunately, it is difficult to pigeonhole the field into traditional academic disciplines. Computational Linguistics is an interdisciplinary topic that has been closer to Linguistics at times, but is currently closer to Computer Science (Engineering), and especially Machine Learning.

To understand the future of our field, we need to understand its past, which we will describe as tribes of researchers migrating through a changing conceptual and socio-economic landscape. The changes are caused by inventions and ideas from outside the field, and also by the field’s own correlated philosophical, methodological, and technical evolution. And the future depends partly on where these research groups are headed now, and partly on what the landscape around them is like.

It is tempting to view this history as a sequence of well-defined stages, defined by a sequence of dominant ideologies about how to define and analyze linguistic patterns. Thus we might list

1. (1950s): Empiricism I—information theory
2. (1970s): Rationalism I—formal language theory and logic
3. (1990s): Empiricism II—stochastic grammars
4. (2010s): Empiricism III—Deep Nets

This description suggests a winner-take-all picture of the field. In fact, the field has always benefited from a give-and-take of interdisciplinary ideas, making room for various combinations of methodologies and philosophies, in different proportions at different times. Logic played a larger role when rationalism was in fashion, and probability played a larger role when empiricism was in fashion, and both logic and probability faded into the background as deep nets gave procedural life to an associationist (rather than statistical) flavor of empiricism. But at every stage, there have been research communities of various sizes inhabiting or exploring different regions of this dynamic landscape, motivated by their various ideological visions, their preferred methodological tools, and their substantive goals. The decades have seen various different communities prosper, decline almost to extinction, and then grow again, waxing and waning in different rhythms. The seeds of the next dominant fashion can always be seen in research communities that seem marginal at a given stage. So after our brief intellectual history, we’ll survey the current landscape, and raise some questions about where the seeds of the next stage might now be found.

In Church (2011), we talked about a pendulum swinging back and forth between Rationalism and Empiricism. There were very few statistical papers in ACL conferences^3^ in 1990. A decade later, there were very few non-statistical papers. Just as Chomsky rebelled against his teachers, our generation returned the favor by rebelling against him and re-introducing statistical papers. The pendulum paper predicted that the next generation would soon rebel against us, as indeed they have. Instead of a return to Rationalism, though, the rebellion took an unexpected turn with the revival of Connectionism. These days, nearly all papers in top venues in computational linguistics (as defined in footnote 1) make use of machine learning, many favoring end-to-end systems over representation (with or without statistics).

Proportions change over time and space. Fads come and fads go. There are booms and there are busts. But good ideas rarely disappear completely, even during winters. Hinton and LeCun believed in nets and kept at it, even when it wasn’t fashionable (Rumelhart et al., 1985; LeCun et al., 1998; Krizhevsky et al., 2012; LeCun et al., 2015). So too, Salton believed in what he was doing, and kept working on projecting text into vector spaces, even when that wasn’t fashionable (Salton et al., 1983; Salton and Buckley, 1988; Salton et al., 1993; Salton and Harman, 2003; Dubin, 2004).

In retrospect, the metaphors of rebellions and pendulums are unfortunate. Sometimes ideas are clashing, especially in the presence of unusually strong personalities like Chomsky, but more often, there is plenty of room for multiple positions to co-exist, sometimes in the very same person. As we will see, Shannon, for example, made important contributions to multiple positions.

Most positions have much to recommend them. At each historical stage, there’s a combination that is better than the best “pure” position. We will refer to this winning combination as “better together.” It is tempting to emphasize differences, but more profitable to emphasize synergies.

### 1.1 Can We Use the Past to Predict the Future?

It is difficult to make predictions, especially about the future,^4^ but it is a good bet that the future will not be like the present. There was considerable relevant history before the 1950s, and there will certainly be more changes in the future.

If you don’t like the weather in New England now, just wait a few minutes.—Mark Twain

As we will see, the times may be ripe for change, though it is hard to say in which direction. The future will likely involve some combination of logic, probability and connectionism, though probably in different proportions from what they are now, or what they were at some point in the past. Since we do not know what will be important in the future, the pendulum paper suggested that we ought to provide today’s students with a broad education that makes room for many topics. That advice is just as appropriate today as it was then, but unfortunately, classes on computational linguistics are removing many topics that we used to teach in order to make more room for modern methods.

Consider BERT (Devlin et al., 2018). Transformer nets have taken the field by storm, nearly 14k citations for this one BERT paper in just two years, with many more citations for other transformer nets: ERNIE (Sun et al., 2020), GPT^5^ (Brown et al., 2020), ELMO (Peters et al., 2018), etc. Given all this excitement over transformers, will there be a future beyond BERT? Probably so, given that history of paradigm changes every few decades.

Sometimes the theory is ahead of the practice,^6^ but these days, the deep net practice is well ahead of the theory. LeCun expressed this fact clearly in a recent podcast.^7^

And the joke is… it works in practice, but does it actually work in theory? And you know, the whole community essentially had this attitude that… it works in practice, but we don’t understand why… so we’re just not going to work on it anymore. In my opinion, this is… looking for your lost car keys under the street light, even though you lost it someplace else.

Elsewhere,^8^ LeCun argued that “Theory often follows invention,” using examples like those in Table 1 to make the case.

**TABLE 1:** Theory often follows invention.

Invention	Theory
Telescope [1608]	Optics [1650–1700]
Steam engine [1595–1715]	Thermodynamics [1824–…]
Microscope (1590)	Cell Theory (1665)
Electromagnetism [1820]	Electrodynamics [1821]
Airplane [1885–1905]	Wing Theory [1907]
Compounds [???]	Chemistry [1760s]
Feedback amplifier [1927]	Electronics […]
Computer [1941–1945]	Computer Science [1950–1960]
Teletype [1906]	Information Theory [1948]

Histories of Artificial Intelligence (AI) often start in 1956 with the naming of the field,^9^ but of course, many of the key ideas are considerably older than the name. As mentioned above, both logic and probability have played an important role in the history of AI. Sometimes logic and probability co-exist in harmony. In fact, Boole’s seminal 1854 book on mathematical logic (Boole, 1854) mentions “probability” in the title: “An Investigation of the Laws of Thought on Which are Founded the Mathematical Theories of Logic and *Probabilities*.” The conceptual split implicit in Boole’s conjunction “Theories of Logic and Probabilities” foreshadowed much of what was to come—and a century later, Claude Shannon made two crucial contributions, one on each side of that divide. His 1937 master’s thesis, which made digital computers possible by translating expressions of Boolean logic systematically into electrical-mechanical terms, was titled “A Symbolic Analysis of Relay and Switching Circuits” (Shannon, 1937). And on the probability-theory side, his 1948 monograph ”A mathematical theory of communication” (Shannon, 1948) introduced the concepts and techniques of information theory based on the assumption that messages are sequences of symbols generated by a stochastic process.

Yet a third concept arose in Ronald Fisher’s 1936 paper “The use of multiple measurements in taxonomic problems” (Fisher 1936), which proposed a method for dividing multivariate measurements into categories based on thresholding a linear combination of the measurement vectors. Although this results in a categorical classification, it is obviously not a logical expression, since it involves arithmetic on numerical measurements. And although Fisher’s method was based on assumptions about the underlying population distributions, the general idea is not limited by any considerations of probability theory—it is just a thresholded inner product. Generalizations of this idea—networks of matrix multiplications interconnected via point non-linearities—have risen to prominence under a variety of names: the “perceptron,” “neural nets,” “parallel distributed processing,” “connectionism,” and most recently “deep learning.” Fisher’s contributions have been extremely influential, and continue to live on to this day, though the 1936 paper was published in a journal on Eugenics, a topic that has fallen out of favor, almost certainly for good reasons.

In 1943, Warren McCulloch and Walter Pitts aimed to bring Boolean logic to neuroscience, in a paper with the title “A logical calculus of the ideas immanent in nervous activity” (McCulloch and Pitts, 1943), arguing that “neural events and the relations among them can be treated by means of propositional logic.” In Stephen Kleene’s 1951 paper “Representation of events in nerve nets and finite automata,” he re-expressed the McCulloch-Pitts system to cover what he called “regular events,” constituting a “regular language” symbolized via what we now call “regular expressions.” This line of work, along with its context of recursive function theory and its development into formal language theory, had enormous influence on linguistics and computer science, but it seems to have been a dead end from the perspective of neural computation. Instead, it was an extension of Fisher’s discriminant analysis that succeeded in that arena, starting with Frank Rosenblatt’s 1958 paper “The perceptron: a probabilistic model for information storage and organization in the brain” (Rosenblatt, 1958).

The phrase “probabilistic model” in Rosenblatt’s title seems to mean only that most of the values used in learning, computation, and storage are gradient rather than categorical, while some binary decisions are made by thresholding: “If the algebraic sum of excitatory and inhibitory impulse intensities is equal to or greater than the threshold of the A-unit, then the A-unit fires, again on an all-or-nothing basis.” The important novelty in this work was a simple iterative method for learning the weights and thresholds needed to make the perceptron compute the right answer.

The result was an enthusiastic ferment of perceptron-inspired speculation, which was dampened by the criticisms in Minsky and Papert’s 1969 book “Perceptron: an introduction to computational geometry” (Minsky and Papert, 1969), which argued that linear perceptrons were too limited for serious applications, and that non-linear networks would be in general impossible to train. Another wave of enthusiasm was sparked by the work summarized in the 1986 book “Parallel distributed processing: Explorations in the microstructure of cognition” (McClelland et al., 1986), which provided an effective method for training multi-layer networks with intermediate point non-linearities, and also presented many impressive (conceptual) applications.

That second wave of connectionist enthusiasm was gradually overcome in the 1990s by the growth of statistical machine learning, epitomized by Vapnik (1995), which promised better results from mathematically more tractable methods. But then a third wave, which we’re still riding, was inspired by the development of better training methods for deep networks, represented by Hinton et al. (2006), and enabled by the spread of powerful GPU-based linear algebra machines.

### 1.2 Connectionism, Associationism and Empiricism

There are also connections between deep nets (connectionism) and associationism in philosophy and psychology, going back to Locke and Hume:^10^

What is Associationism? … pairs of thoughts become associated based on the organism’s past experience…. [A] basic form of associationism (such as Hume’s) might claim that the frequency with which an organism has come into contact with Xs and Ys in one’s environment determines the frequency with which thoughts about Xs and thoughts about Ys will arise together in the organism’s future.

Associationism’s popularity is in part due to how many different masters it can serve…. [A]ssociationism … has also been closely aligned with a number of different doctrines over the years: empiricism, behaviorism, anti-representationalism.

Like associationism, deep learning is a particular take on empiricism. And like deep learning (and in contrast to statistics and probability theory), associationism has never really connected with mathematics. What’s different, obviously, is that big fast cheap computers make it possible for versions of associationism to be implemented and tested, more elaborately and practically than the philosophers or the rat-runners could.

People think about what they can afford to think about. As LeCun pointed out in a recent talk on the history of nets, “there is something weird … when hardware is too slow … software is not readily available—experiments are not reproducible because of lack of open source—people will find ways to dismiss good ideas.”^11^

In private discussions with others (personal communication), it has been suggested that the community was slow to appreciate the merits of deep nets because they had “no way to know or validate whether it is a good idea of not.” Recent emphasis on evaluation and shared benchmarks has been good for deep nets.

### 1.3 Representation vs. End-To-End

A classic question in AI is the role of representation. Minsky thought representation was the key to AI (Minsky, 1961; Minsky and Papert, 1969) and never warmed to neural nets.^12^

A number of others have criticized end-to-end systems. Tukey (1977) advocated an alternative which he called exploratory data analysis (EDA). EDA emphasizes a role for a human in the loop. Chomsky (1959) objected to the use of black boxes in Skinner’s Behavorism.

As mentioned above, end-to-end systems have become extremely popular recently. Mercer was an early advocate of end-to-end approaches (Church and Mercer, 1993). This approach led to the famous quote, “Every time I fire a linguist, the performance of our speech recognition system goes up.”^13^ The IBM group used the term “self-organizing systems” in papers on speech recognition (Jelinek, 1990) and machine translation (Brown et al., 1988). After leaving IBM, many of the same people probably used similar methods in hedge funds and politics, though they do not talk so much about those use cases (Zuckerman, 2019). The term “self-organizing” dates back to the 1950s (Von Foerster, 1959), if not earlier.

More recently, LeCun made a nice case for the end-to-end approach in a podcast.^14^

All of AI relies on representations. The question is where do those representations come from? So, uh, the classical way to build a pattern recognition system was … to build what’s called a feature extractor … a whole lot of papers on what features you should extract if you want to recognize, uh, written digits and other features you should extract if you want to recognize like a chair from the table or something or detect…

If you can train the entire thing end to end—that means the system learns its own features. You don’t have to engineer the features anymore, you know, they just emerge from the learning process. So that, that, that’s what was really appealing to me.

One concern with the end-to-end approach is that it encourages young researchers to focus too much on some things (network architecture and training methods), and not enough on other things (insights from literature from a wide range of relevant disciplines, about both methodology and content). One of the themes of this paper is that we ought to provide the next generation a broad education because we do not know what will be important next—unfortunately, courses are under increasing pressure to make room for currently popular methods at the expense of traditional topics.

There is considerable discussion of black boxes and glass boxes in software engineering (Boehm and Papaccio, 1988). In software engineering, though maybe not in machine learning, it is generally accepted that there should be plenty of room for both system testing (black boxes) as well as unit testing (glass boxes). Standard benchmarks in computational linguistics such as papers with code^15^ and GLUE^16^ (Wang et al., 2018) tend to emphasize system testing, though Ribeiro may be successful in advocating more use of unit testing (Ribeiro et al., 2016; Ribeiro et al., 2020).

Although it might be possible to explain why a net does what it does without looking inside the box, a common motivation for looking inside the box is explanation. Nets work surprisingly well most of the time, but sometimes they work surprisingly badly.

Although research on “end-to-end” neural TTS has produced impressive demonstrations, our own results suggest that they will make embarrassing errors when applied to arbitrary text, and such errors would be hard to fix in an end-to-end system (Zhang et al., 2019).

There has been considerable interest recently in interpretability with tutorials on that subject at recent conferences such as NeurIPS-2020,^17^ EMNLP-2020 (Wallace et al., 2020),^18^ and ACL-2020 (Belinkov et al., 2020).

### 1.4 An Example of Representations: Semantic Nets

It is natural to model words (and concepts) as nodes in a graph, with edges representing relations such as synonyms, antonyms and hypernyms (*car* is a *vehicle*). There are many names for such graphs: knowledge graphs, semantic networks, ontologies, etc. These graphs can be used to represent lexical knowledge and/or world knowledge (facts about the world that go beyond linguistics). Examples of semantic nets: WordNet^19^ (Miller et al., 1990), CYC (Lenat, 1995), Freebase/Wikidata (Bollacker et al., 2008). Many of these resources were originally developed for English. Some have been extended to address other languages.^20^ BabelNet 4.0^21^ (Navigli and Ponzetto, 2012) supports 284 languages. Many of these ontologies can be downloaded for free. All have been successful (at least in terms of citations).

Some of these projects are more ambitious than others. CYC is particularly ambitious, perhaps too ambitious. WordNet is less ambitious; the scope of this project was constrained by the relatively modest budget. Many projects of this kind, such as Murray’s Oxford English Dictionary (Murray, 2001) and CYC, have a way of consuming all available resources (and then some). Projects are like a gas; they expand to fill the container. WordNet (and Unix) succeeded, largely because “Less is more” (McMahon et al., 1978).

According to the documentation on WordNet,^22^

The main relation among words in WordNet is synonymy, as between the words shut and close or car and automobile. Synonyms—words that denote the same concept and are interchangeable in many contexts—are grouped into unordered sets (synsets).

The WordNet database assigns words to 117k synsets. Many words are ambiguous, and are assigned to two or more synsets. Synsets are connected to one another by relations such as hypernymy (is-a) and meronymy (part-whole). There are additional connections for verbs and adjectives.

Many resources have been built with volunteer labor: Freebase, Wikipedia and Murray’s Oxford English Dictionary. If one wants to be more ambitious than WordNet, it may not be feasible to pay a full-time staff to do the work by hand. Universities can use student labor to reduce labor costs. The gig economy offers opportunities to reduce labor costs even more (Zaidan and Callison-Burch, 2011), raising various ethical questions (Hara et al., 2018). Savage gave a couple of recent talks titled “A Future of Work for the Invisible Workers in A.I.,” at NeurIPS-2020 and elsewhere,^23^ calling attention to just how much modern methods depend on gig workers, as well as possibilities for exploiting these workers.

In the future, it may be possible that machine learning methods such as knowledge graph completion (KGC) could make it more feasible to construct linguistic resources such as WordNet, CYC and Freebase. KGC starts with 〈h,r,t〉 triples, where *h* (head) and *t* (tail) are nodes in a graph. There are standard benchmarks such as WN18RR and FB15k-237 which are derived from WordNet and Freebase, respectively. There are 11 relations in WN18RR, but most of the edges fall into just two relations: (a) is-a, or (b) derivationally-related-form. (The name of relation (b) is a bit misleading; in practice, it combines synonyms and regular inflection.) The benchmark splits edges randomly into train, validation and test sets. The task is to learn a model from the training set, and predict the edges in the held-out test set. Surveys (Nguyen, 2017) describe a number of methods such as Trans[DEHRM] that perform well on these benchmarks. Many of these methods are available for download in a convenient python package:^24^ Yu et al. (2019).

Despite impressive results, it is unclear if KGC methods will improve coverage of resources such as WordNet and Freebase (Church, 2020). The benchmark task appears to be evaluating lexical and/or world knowledge, but one can do remarkably well on this task by counting cards, as in bridge. Relations like synonymy are (nearly) equivalence relations. Pairs of words in the same synset are likely to be connected, and pairs of words in different synsets are unlikely to be connected. In the KGC game, edges (connections) are assigned to three sets: training, validation set and test. We can think of this assignment like dealing cards in bridge, except there are three hands in the KGC game, as opposed to four hands in bridge. Bridge players guess which cards are in which hand by the process of elimination and circumscription. That is, they know how many cards there are, and they know that each card must be in one and only one hand. So too, similar card counting methods are remarkably effective for guessing which edges are in which set in the KGC game. One can guess how many edges there are by forming the transitive closure of the edges in the training set. If an edge is in the transitive closure, but not in the training set, then it is likely to be in the test set. There are more interesting variants of the KGC game that could be more useful in practice, as we will see when we discuss OOVs.

Recently, there has been considerable interest in static embeddings [Word2Vec (Mikolov et al., 2013c), GloVe (Pennington et al., 2014)] and contextual embeddings [BERT (Devlin et al., 2018), ERNIE (Sun et al., 2020), GPT (Brown et al., 2020), ELMO (Peters et al., 2018)]. Pre-trained embeddings can be downloaded in many languages^25^ and many domains including medicine and twitter (Lee et al., 2020; Nguyen et al., 2020). In contrast to WordNet, CYC, Freebase, etc., embeddings can be learned by unsupervised methods from text with no need for manual labor and/or annotations (supervision).

Static embeddings represent words as vectors. The similarity of two words is simply the cosine of the vectors for the two words. Levy and Goldberg (2014) suggest word2vec cosine is similar to Pointwise Mutual Information (PMI) (Church and Hanks 1990).

Contextual embeddings are widely viewed as an improvement over static embeddings. The first layer of a contextual embedding is essentially a static embedding, but deeper layers take advantage of context so it is no longer the case that two mentions of the same word will be represented by the same vector. Contextual embeddings are deep nets with typically a dozen layers.

Embeddings (and PMI) depend on Firth (1957): “You shall know a word by the company it keeps.” Collocation-based methods (Word2Vec, GloVe, PMI) tend to assign high similarity scores to words that can be compared (or contrasted), including both synonyms as well as antonyms. It makes sense that synonyms should be considered similar, but it is unfortunate that antonyms are also considered similar.

In general, tall skinny matrices do not work well with modern GPUs. Embeddings are V×K matrices, where *V* is the size of the vocabulary and *K*, the length of the word2vec vectors, are referred to as hidden/latent/internal dimensions. Typically V≫K where *V* is at least 30,000 and K≈300. Large vocabularies (V≫30,000) are particularly challenging for deep nets.

Vocabularies of 30,000 words cannot be expected to cover most corpora. The remaining words are referred to as OOVs (out of vocabulary) words. Word pieces are often used for OOVs. BERT provides the following motivation for word pieces:

“Using wordpieces gives a good balance between the flexibility of single characters and the efficiency of full words for decoding, and also sidesteps the need for special treatment of unknown words.” (Devlin et al., 2018).

Subwords are based on BPE (byte pair encoding), which borrows ideas from information theory to learn a dictionary of word pieces from a training corpus. Word pieces are used for a variety of applications: speech (Schuster and Nakajima, 2012), translation (Wu et al., 2016), as well as tasks in the GLUE benchmark such as: sentiment, paraphrase and coreference.

Linguists will find subwords counter-intuitive. Given the unknown OOV (out-of-vocabulary) word, *unidirectional*, BERT produces five tokens: *un ##idi ##re ##ction ##al*, as opposed to the more intuitive analysis: *uni-directional*.

One might be concerned that subwords (and BERT) may have been designed for languages like English with particular types of spelling systems, relatively fixed word order and Indo-European etymology. In fact, pre-trained BERT-like models are available in many languages (see footnote 25). There is considerable interest in BERT-like models for Chinese, a commercially important language that is very different from English in a number of ways including spelling, word order and etymology.^26^

Subwords offer two benefits: (1) reducing *V* reduces computation, and (2) portability. Methods that depend on manually created resources (grammars, morphological rules, annotated corpora and dictionaries of morphemes) tend to be expensive to port to new languages and new domains. Unsupervised methods such as subwords avoid these costs.

That said, it ought to be possible (and useful) for unsupervised methods to capture the kinds of generalizations that linguists are interested in. Consider word formation processes. BERT has two processes, one for words in the vocabulary of *V* words such as *directional*, and another for other words such as *unidirectional*. It ought to be possible for unsupervised methods to introduce a third process (word formation processes) to connect the dots between known words and “almost known” words so that it would be easier to capture generalizations involving sound and meaning (Jakobson et al., 1978).

KGC methods do not currently address almost known words, but this would be an excellent opportunity for KGC. If we have an OOV such as *unidirectional* with no links in the knowledge graph, but we can infer that *unidirectional* is near *directional*, can we infer much of the sound (phonemes and stress) and meaning (ontology and/or embeddings) for the OOV from the known word plus the word formation process. That is, if we have the meaning (ontology and/or embeddings) for the known word, *directional*, and we have the meaning for lots of pairs of words, 〈x,uni+x〉 that are connected by the *uni-*word formation process, can we infer the meaning of the almost known word, *unidirectional*. Generalizing the KGC game to almost known words would be useful in practice since many OOVs are near known words.

## 2 Pros and CONS

### 2.1 Pros: Successes

There is no question deep nets have produced dramatic improvements in performance in recent decades. There are web sites (see footnote 15) that track SOTA (state of the art) progress over time on many (228) tasks in natural language processing, and hundreds more in related areas in speech and vision and more. These timelines make it clear that there has been considerable progress on almost all of these tasks, and that modern methods (neural nets) are largely responsible for the bulk of this progress. The 2018 Turing Award recognizes Hinton, LeCun and Bengio for this amazing accomplishment.

“Deep neural networks are responsible for some of the greatest advances in modern computer science, helping make substantial progress on long-standing problems in computer vision, speech recognition, and natural language understanding,” said Jeff Dean, Google Senior Fellow and SVP, Google AI. “At the heart of this progress are fundamental techniques developed starting more than 30 years ago by this year’s Turing Award winners, Yoshua Bengio, Geoffrey Hinton, and Yann LeCun. By dramatically improving the ability of computers to make sense of the world, deep neural networks are changing not just the field of computing, but nearly every field of science and human endeavor.”^27^

The ACM calls out three accomplishments for each of the three winners:

1. Hinton: Backpropagation, Boltzmann Machines, Improvements to convolutional neural networks
2. Bengio: Probabilistic models of sequences, High-dimensional word embeddings and attention, Generative adversarial networks
3. LeCun: Convolutional neural networks, Improving backpropagation algorithms, Broadening the vision of neural networks

There is no question that these methods work extremely well, not only on academic benchmarks, but also on commercially important applications. Dale (2019) recently published a survey of 25 years of commercialization of NLP in the Journal of Natural Language Engineering, as part of their celebration of the 25th year of that journal. He compared the current state to a 1995 survey by the first author (Church and Rau, 1995).

It is much easier to write a survey of successes today than it was in 1995. Recall that the web was just about to take off in 1995. That was before Google^28^ and BAT (Baidu, Alibaba, Tencent).^29^ In retrospect, the economic miracle in China and India has been miraculous, but at the time, the miracle was not as obvious as it is today, especially to the rest of the world. Similar comments apply to Silicon Valley, as well, as evidenced by real estate prices. Almost no one could have guessed just how important our field would become, with a few notable exceptions (Saxenian, 1996).

These days, everyone knows about AI technologies. Most people have considerable experience with web search, speech recognition, speech synthesis (in mapping applications), face recognition and more. But that was not always the case. There was a time, not so long ago, when our friends and family would ask us what we were working on, and they would respond with strange looks.

These successes were quite unexpected, at least a couple of decades ago. See Moore (2005) for a comparison of two surveys of speech experts. Moore reported in his Figure 1 a striking similarity between the survey responses in 1997 and 2003. In both surveys, speech experts thought it would take another decade for various results to be delivered. In other words, there was little evidence of progress between 1997 and 2003 (subjective or objective). After an additional survey in 2009, Moore concluded Moore (2011):

[T]he future appears to be generally no nearer than it has been in the past. However, on a positive note, the 2009 survey confirmed that the market for speech technology applications on mobile devices would be highly attractive over the next ten or so years.

The third time the experts predicted that such and such was only a decade away, they got it right. Perhaps they knew what was coming, or perhaps, if you predict just-another-decade often enough, you will eventually get it right.

Recall that cell phones were considered expensive at the time. Smart phones were just beginning to happen. The first smart phone came out in 2007.^30^ SIRI was released in 2011.^31^ Smart speakers came out a few years later.^32^ Some of these improvements came about because of amazing advances in hardware and Moore’s Law (Schaller, 1997), but many of these improvements came about because of advances elsewhere, as well.

These days, smart phones and smart speakers make it much easier to think about all sorts of possible applications. As mentioned above, LeCun pointed out (see footnote 11) that people think about what they can afford to think about. When hardware is too slow, and results are hard to compare and reproduce, people will find ways to dismiss good ideas. Evaluation was not as widely accepted back then. Computing power was limited. No GPUs. No compute clusters. No AWS (Amazon Web Services).^33^ Data sets were hard to come by. The LDC (Linguistic Data Consortium)^34^ was founded just a few years before (1992), and the bulk of their collection was not available at the time. ELRA (European Language Resources Association)^35^ was created soon thereafter (1995). Many of these factors (and more) contributed to today’s successes.

### 2.2 Cons: Alchemy

Despite these successes (or perhaps because of these successes), the time seems to be ripe for change. Is end-to-end performance on a benchmark all there is, or should we be concerned with other issues as well, such as understanding? Explanation? Interpretation? Causality? Domain Transfer? Ethics?

Some people take one side of the Alchemy debate and others take the other. Some debates hinge on the content of the arguments. Sometimes rhetoric trumps content. Sometimes the audience comes to the debate with their mind already made up. If the prior is strong enough, both content and rhetoric are moot.

Most people have strong priors on the Alchemy debate. There may not be any persuadable voters left on the Alchemy debate, but nevertheless, the base still loves to hear their side score (pointless) points. Preaching to the choir may not swing elections, but it sells lots of newspapers.

The Alchemy argument comes up whenever practice appears to be ahead of theory. This debate came up recently in Rahimi’s NIPS-2017 Test of Time Award talk: “Machine learning has become Alchemy.”^36^ This debate grants that AI works well in practice, but raises concerns about the ever-growing space of ill-understood techniques (so-called model zoo).

The Alchemy argument was also used as an insult in the 1960s, when AI did not work well, either in theory or in practice. Dreyfus (1965) ^37^ argued that human intelligence and expertise depend primarily on unconscious processes rather than conscious symbolic manipulation, and that these unconscious skills can never be fully captured in formal rules.

For more on the recent alchemy debate, see “LeCun vs Rahimi: Has Machine Learning Become Alchemy?”^38^:

According to Rahimi, machine learning research and alchemy both work to a certain degree. Alchemists discovered metallurgy, glass-making, and various medications; while machine learning researchers have managed to make machines that can beat human Go players, identify objects from pictures, and recognize human voices…

“We are building systems that govern healthcare and mediate our civic dialogue. We would influence elections. I would like to live in a society whose systems are built on top of verifiable, rigorous, thorough knowledge, and not on alchemy,” said Rahimi.

That triggered Facebook Director of AI Research Yann LeCun, who responded to Rahimi’s talk the next day, saying the alchemy analogy was “insulting” and “wrong.” “Criticizing an entire community (and an incredibly successful one at that) for practicing ‘alchemy,’ simply because our current theoretical tools haven’t caught up with our practice is dangerous.”

LeCun had more time to respond at a debate on interpretability at NIPS-2017^39^

Ok now, which one do you want? The one that is explainable, or the one that actually works?

The first author took the other side on this debate in “I did it, I did it, I did it, but … ” (Church, 2017).

There has been a trend for publications to report better and better numbers, but less and less insight. The literature is turning into a giant leaderboard, where publication depends on numbers and little else (such as insight and explanation). It is considered a feature that machine learning has become so powerful (and so opaque) that it is no longer necessary (or even relevant) to talk about how it works. Insight is not only not required any more, but perhaps, insight is no longer even considered desirable.

As for reliability and explainability, the narrative is that neural nets are black boxes that do not always work. It is hard to know when they will work, and why they do what they do. LeCun addresses these concerns in various places such as this^40^ with:

it [explanation] is not as useful as most people think… the vast majority of decisions made by machine learning systems don’t require explanations … but there are certain decisions… in the legal domain, for exmaple… there has been a bit of myth that neural nets are black boxes … we can’t understand what’s inside … that’s not true … we can … we can look at all the variables … we can do sensitivity analyses … all kinds of techniques … the reason they are not used is that they are not that useful”

Some people suggest explanation is not important, and others disagree. The first author taught a reading class, where there was considerable interest in explainable AI and ethics. The segment on a recent best seller, “Weapons of Math Destruction” (O’Neil, 2016),^41^ was one of the more influential segments, especially among women and minorities. Several of them chose to write term papers on that subject.

As mentioned above, the times seem to be ripe for a change. It is likely that explanation and ethics will be taken more seriously going forward than they have been in the past. When we write about these questions (Church, 2017), metrics on views respond favorably;^42^ the audience is clearly concerned about explainable AI and ethics.

Transparency is good and opacity is bad. A recent best seller, Weapons of Math Destruction, is concerned that big data (and WMDs) increase inequality and threaten democracy largely because of opacity. Algorithms are being used to make lots of important decisions like who gets a loan and who goes to jail. If we tell the machine to maximize an objective function like making money, it will do exactly that, for better and for worse. Who is responsible for the consequences? Does it make it ok for machines to do bad things if no one knows what’s happening and why, including those of us who created the machines?

LeCun was given even more time to respond to the alchemy criticism at an 2019 IAS event “Deep Learning: Alchemy or Science?”^43^ The IAS event set up the debate with:

Deep learning has led to rapid progress in open problems of artificial intelligence—recognizing images, playing Go, driving cars, automating translation between languages—and has triggered a new gold rush in the tech sector. But some scientists raise worries about slippage in scientific practices and rigor, likening the process to “alchemy.” How accurate is this perception? And what should the field do to combine rapid innovation with solid science and engineering.

LeCun used his time to describe a long history of successes.^44^ There is no question that deep nets are producing impressive results, but nevertheless, there is a sense in the community that the time might be right for the Next AI Campaign, as will be discussed in section 3.1.

The debate is often seen as a contest between engineering and science, with LeCun on the side of engineering and others such as the Psychologist Josh Tenenbaum on the other side. LeCun cares deeply about making machines that work, whereas Tenenbaum would like to understand common sense and learning, especially in young children (and animals).

That said, this characterization of the debate is probably overly simplistic. LeCun is, in fact, very sympathetic to Tenenbaum’s position:^45^

But you know, I’m perfectly ready to throw probability under the bus… What is the obstacle to AI? Why is it that we don’t have machines that can navigate the world as well as a cat… Why is it that we don’t have machines that learn language as … well as kids… You know the punch line is… our AI systems need to learn models of the world… it is very similar to what Josh was saying

As suggested above, the time seems to be ripe for change. LeCun is well aware of various limitations of currently popular methods:^46^

There is a limit to what you can apply deep learning to today due to the fact that you need a lot of labeled data to train them. And so, it’s only economically feasible when you can collect that data and you can actually label it properly. Uh, and that’s only true for a relatively small number of applications.

it works great for speech recognition… but it doesn’t work for all kinds of stuff… where it’s very expensive to collect data, like medical images for example…. if you want to train a system to hold a dialogue with someone, you cannot just collect the training set and…

LeCun has a nice way of making such points extremely crisply: “the revolution will not be supervised.”^47^

## 3 Perspectives From Funding Agencies Around the World

### 3.1 DARPA’s ”AI Next” Campaign

Funding agencies like DARPA have a mission to change the world in fundamental ways that will stand up to the test of time. When Licklider first established DARPA’s IPTO (Information Processing Techniques Office) in 1962, the mission was:^48^

[To] create a new generation of computational and information systems that possess capabilities far beyond those of current systems. These cognitive systems—systems that know what they’re doing:

1. will be able to reason, using substantial amounts of appropriately represented knowledge;

2. will learn from their experiences and improve their performance over time;

3. will be capable of explaining themselves and taking naturally expressed direction from humans;

4. will be aware of themselves and able to reflect on their own behavior;

5. will be able to respond robustly to surprises, in a very general way.

IPTO has made a number of extremely important contributions including time-sharing and the internet. This office has also supported much of the work on HLT (Human Language Technology). Web search is now a Fortune 500 business. Speech is not yet as successful as web search, but (nearly) all of our friends and family have considerable experience with the technology. There was a time when we would have to explain to people what we did and how we were working on making machines talk and listen, but that was a long time ago.

So, how is IPTO doing on that mission, and what should it do next? At the DARPA “AI Next” conference, it was widely understood that there have been great advances over the years, especially recently with Machine Learning and Deep Nets. But, it was also understood that we do not need more cat detectors. While machine learning works really well if the test set is like the training set, the methods are not very robust to surprises. Much of the mission statement above remains a work in progress, especially the last line on surprises. DARPA (and other funding agencies) have encouraged considerable work on surprise languages, low resource languages, transfer learning, zero- (and few-) shot learning, and more.

So what does DARPA plan to do next?

DARPA is now investing more than $2 billion in the next generation of AI through its initiative, AI Next. Advances will not only require simultaneous and coordinated progress in knowledge representation and reasoning, machine learning, human language technology, and vision and robotics, but also in the tight integration of each component to realize trustworthy intelligent systems able to operate autonomously or team with humans. (Fouse et al., 2020, p. 4)

DARPA is encouraging the field to start a new third wave in AI. They recently organized a conference^49^ where it was made clear, at least in hallway conversations (personal communication), that we do not need more “cat detectors.” Videos of many of the talks (though unfortunately, not the hallway conversations) are available online.^50^ Many of these talks encouraged work that combines theory (generative models) and practice (pattern matching).

The first slide of Tenenbaum’s keynote said:Where is the gap?

• Intelligence is not just about pattern recognition.
• It is about modeling the world…
  • explaining and understanding what we see.
  • *imagining* things we could see but haven’t yet
  • *problem solving* and *planning* actions to make those things real.
  • *building new models* as we learn more about the world.

The press^51^ talks about three waves.

1. Expert Systems: “if this, then do that”
2. Machine Learning: Siri, face recognition, etc.
3. AI Next: TBD (to be determined)

It is pretty clear what the first two waves were, but the next wave is more of a work in progress:

But the emphasis is on the third wave, which is all about broadening the application of AI, or adding what you might call “contextual reasoning,” or even common sense. In situations an AI system has never encountered or been explicitly trained for, can it learn to pick up clues from the environment and come to accurate conclusions? DARPA is betting yes, says Pierce.

The first wave, expert systems, was programmed by hand, with no capacity for learning. There were some notable successes (such as chess), but coding everything by hand turned out to be expensive and hard to scale.

The current second wave produced numerous impressive successes such as Siri, face recognition and image classification. But machine learning has run into various limitations: requirements on training data, robustness and adaptation to changing contexts, etc. We are seeing better and better numbers on benchmarks, but benchmarks often underestimate real world difficulties. In benchmarks, the test and train are often drawn from the same population. Such idealizations lead to Godfrey’s Gap, where performance on benchmarks is unrealistically rosy (Church and Hestness, 2019). On problems that matter to the sponsor, performance can be much worse because we have no idea what real users will do, but it probably won’t be well covered by the training set.

Evaluations tend to focus on typical (or average) case performance, but to achieve acceptable robustness, we probably need to do more to handle novelty and adversarial attacks. There are many tasks that are so easy that even a child can do them, but they are beyond the capabilities of second wave systems.

The third wave is described as follows here:^52^

The past few years have seen an explosion of interest in a sub-field of AI dubbed machine learning that applies statistical and probabilistic methods to large data sets to create generalized representations that can be applied to future samples. Foremost among these approaches are deep learning (artificial) neural networks that can be trained to perform a variety of classification and prediction tasks when adequate historical data is available. Therein lies the rub, however, as the task of collecting, labelling, and vetting data on which to train such “second wave” AI techniques is prohibitively costly and time-consuming.

DARPA envisions a future in which machines are more than just tools that execute human-programmed rules or generalize from human-curated data sets. Rather, the machines DARPA envisions will function more as colleagues than as tools. Towards this end, DARPA research and development in human-machine symbiosis sets a goal to partner with machines. Enabling computing systems in this manner is of critical importance because sensor, information, and communication systems generate data at rates beyond which humans can assimilate, understand, and act. Incorporating these technologies in military systems that collaborate with warfighters will facilitate better decisions in complex, time-critical, battlefield environments; enable a shared understanding of massive, incomplete, and contradictory information; and empower unmanned systems to perform critical missions safely and with high degrees of autonomy. DARPA is focusing its investments on a third wave of AI that brings forth machines that understand and reason in context.

The AI Next Conference announced a number of efforts to encourage more interdisciplinary combinations of theory (generative models) and practice. There were talks from a wide range of disciplines including psychology, physics and chemistry. Applications included.

1. predicting what a kid will do next (in a video),
2. predicting which way a stack of blocks will fall (when someone hits the table), and
3. finding better (faster, cheaper and more robust) ways to synthesize chemical models.

DARPA is convinced that we need more than simple pattern matching to make progress on surprises (where the test cases are not like anything in the training set).

### 3.2 Perspectives From Europe and Asia

In America, there are strong connections between academic funding and the department of defense and industry, dating back to WW II (Bush, 1945; Kevles, 1977).^53^ There have been connections between academic funding and industry elsewhere, such as the Japanese Fifth Generation Project (Feigenbaum and McCorduck, 1983), but the connection between academic funding and defense appears to be uniquely American. Funding has been relatively strong over many decades, perhaps because budgets for defense are relatively large in America.

Priorities are different in different places. Government(s) in Europe are trying to build a community, whereas in America, they are trying to rock the boat. In Chinese industry, there is an emphasis on bold initiatives and metrics/milestones.

As usual, much is in a state of flux. Brexit will likely complicate matters in Europe. Funding in Britain will depend more on British programs.^54^ Going forward, the British are less likely to collaborate with Europe, and the Swiss are more likely to collaborate.^55^

A number of efforts in Europe are described here.^56^ In 2020, the European Commission invested €50m in these efforts, after an initial investment of €20m for the creation of AI4EU, the AI-on-Demand-Platform that allows the exchange of AI tools and resources across Europe.^57^ Efforts in Europe are similar to efforts elsewhere, though there is more emphasis in Europe on inclusiveness and diversity (e.g., “79 partners from 21 countries”).

Funding for AI has been increasing over time almost everywhere. That said, it is common for one region of the world to argue that it should receive even larger funding increases because of investments elsewhere. Feigenbaum and McCorduck (1983) used this argument to suggest that America should invest more in AI because of investments in Japan in the 1980s. More recently, MIT’s alumni magazine, Technology Review, suggested that America should invest more in AI because of investments in China.^58^ It is interesting to see similar arguments in Europe, based on investments in America:^59^

Over the past 3 years, EU funding for research and innovation for AI has risen to €1.5 billion, i.e., a 70% increase compared to the previous period.

However, investment in research and innovation in Europe is still a fraction of the public and private investment in other regions of the world. Some €3.2 billion were invested in AI in Europe in 2016, compared to around €12.1 billion in North America and €6.5 billion in Asia.^60^ In response, Europe needs to increase its investment levels significantly.

Much of this white paper discusses data privacy. Data privacy is a concern everywhere, but especially in Europe. It has been suggested, at least in parts of America, that data privacy is less respected in Asia, though in fact, American industries have been relatively successful in taking advantage of the upside opportunities, and appear to be less concerned with potential downside risks. China is adopting regulation^61^ that is similar to regulation in Europe such as GDPR (General Data Protection Regulation).

Funding levels have consequences. When we first published in ACL in the 1980s, the ACL was much smaller than it is today, but it was also much more American. European participation increased with the creation of the European Chapter (EACL), which held its first meeting in 1983. But more recently, participation has increased considerably from Asia, and especially from China. In 2020, there were about as many submissions from China as from the US.^62^ Both countries submitted about 1,000 papers each, with “about”^63^ half as many submissions from the European Union, and another 1,000 papers from the rest of the world. The press in Asia is tracking statistics such as government investments and numbers of publication by region:^64^

AI leadership had in recent years become a two-horse race between China and the US, said Anthony Mullen, director of research at advisory firm Gartner. At the European Conference on Computer Vision … , China ranked first in terms of accepted academic papers. Even if you combine [each European countries’ accepted academic papers], it still won’t be among the very top”

The perspective from Europe is similar, though more positive toward Europe. Annoni et al. (2018) ^65^ report a number of different statistics in Figures 3, 4, 6, 7 and 9. Most of these statistics support the conclusion that Europe is bracketed between the Unites States and China.

## 4 Paths Forward

In Church (2011), we talked about the motivations behind the revival of empiricism in the 1990s.

What motivated the revival of empiricism in the 1990s? What were we rebelling against? The revival was driven by pragmatic considerations. The field had been banging its head on big hard challenges like AI-complete problems and long-distance dependencies. We advocated a pragmatic pivot toward simpler more solvable tasks like part of speech tagging. Data was becoming available like never before. What can we do with all this data? We argued that it is better to do something simple (than nothing at all). Let’s go pick some low hanging fruit. Let’s do what we can with short-distance dependencies. That won’t solve the whole problem, but let’s focus on what we can do as opposed to what we can’t do. The glass is half full (as opposed to half empty).

The situation is different today. Conferences are huge (perhaps too huge). Much of the work is incremental (perhaps too incremental). How can anyone make a difference?

The answer depends on individual interests, skills, and opportunities—but people with different roles in the system face systematically different parts of the landscape. Funders have different challenges from senior researchers, who face different challenges from young researchers, and ditto for investors, managers, etc.

For many people, the temptation is still to reach for low hanging fruit. But we feel that this is probably not the answer in today’s reality. We suggest the following paths forward for funding agencies, managers in industry, senior researchers and younger researchers.

### 4.1 Paths Forward for Funding Agencies

We have described DARPA’s vision of “AI Next,” which is based on the premise that we need more than just ever-larger datasets fed into new varieties of ever-larger networks. A central theme is partial rejection of the radical empiricism of much recent AI research, in favor of systems that do not need to learn, over and over again, the core properties of their domains. These are things like gravity and optics in vision systems, or duality of patterning and hierarchical compositionality in language.

There are some important applications areas, like education, law, and medicine, that have still not really learned the “big data” lesson that image, speech, and language processing internalized in the 1990s. But these areas have special problems with the unpredictable brittleness and lack of explainability of currently fashionable AI, and so they too have reasons to look beyond the radical empiricism of end-to-end approaches.

### 4.2 Paths Forward for Managers in Industrial Research

It is easy to advise funding agencies to look beyond currently low-hanging fruit, since a key part of their mission is to look a decade or more into the future. It is a harder choice for managers in industrial research labs, who are under pressure to produce practical results, often on shorter time scales.

But often, mixing in less-fashionable (and even old-fashioned) methods leads to better results. Consider Google’s approach to text normalization mentioned in Section 1.3. The extreme end-to-end view of speech synthesis (Wang et al., 2017) may be sexy and attractive to researchers, and could well turn out to be the path forward in the long term, but in the short-term, it may be more prudent and pragmatic to take a hybrid approach that combines the best of the old with the best of the new. End-to-end nets produce amazing results when they work, but they can also produce embarrassing mistakes (that only a computer could make). Zhang et al. (2019) advocate a hybrid of nets with covering grammars (traditional finite-state methods),^66^ to keep nets in bounds, and avoid embarrassing mistakes.

Longer-term, managers in industrial research should also keep an eye on initiatives like AI Next, since some of the new approaches may start to become practical (and therefore fashionable) sooner rather than later.

### 4.3 Paths Forward for Senior Researchers

Many senior researchers are perceiving the same need for new directions that we have been talking about. David Forsyth (personal communication) likes to show a video of a donkey in an awkward position.^67^ The donkey is pulling a cart, but when some of the load falls off the back of the cart, the donkey is lifted into the air and could not reach the ground. As a number of people slowly removes the load from the cart, it becomes more and more clear how the video will end. Even the donkey can predict that he will eventually get back on his feet and live happily ever after. The challenge for our AI technology is to come up with ways to predict the future. Obviously, it is hard to make predictions, especially about the future, but even so, it is embarrassing if a donkey can do it (and we cannot).

Forsyth’s donkey video makes the same basic point as Josh Tenenbaum’s keynote at the AI Next conference.^68^ Tenenbaum pointed out that kids can do things that go way beyond what machines can do. He showed a video of an adult trying to open a door unsuccessfully in front of a 1.5 year old kid. The kid could easily figure out what the adult was trying to do, and showed the adult how to open the door. Tenenbaum pointed out that machines can’t do this, and that current machine learning approaches are unlikely to get us there.

People learning new concepts can often generalize successfully from just a single example, yet machine learning algorithms typically require tens or hundreds of examples to perform with similar accuracy. People can also use learned concepts in richer ways than conventional algorithms—for action, imagination, and explanation (Lake et al., 2015).

Tenenbaum, like Forsyth and DARPA and many others, have been encouraging researchers to think more about zero-shot, one-shot and few-shot learning. Tenenbaum’s keynote called out the Omniglot data set^69^ for developing more human-like learning algorithms. It contains 1,623 different handwritten characters from 50 different alphabets. Each of the 1,623 characters was drawn online via Amazon’s Mechanical Turk by 20 different people. Each image is paired with stroke data, a sequences of [x,y,t] coordinates with time (t) in milliseconds. In a more recent report summarizing three years of work on Omniglot, Lake et al. (2019) point out that there has been notable progress, especially on one-shot classification.

Tenenbaum advocates a combination of theory and practice similar to a GAN (Generative Adversarial Network)^70^, in which a theoretical model that generates future possible worlds—using a system that understands (or at least incorporates) the basic physics of the situation—trains an empirical subsystem for predicting and evaluating such futures based on (incomplete) knowledge of the present, such as what can be learned from image analysis.

Traditional “cat detectors” work well when they work. As discussed in Section 3.1, we should celebrate these short-term successes, but there is a risk that such celebrations could distract the field away from what really matters in the long-term. Despite amazing recent progress, the field still has considerable work to do on DARPA’s 1962 mission statement, especially the last line: “to respond robustly to surprises.” Nearly all senior investigators and funders are aware of these issues, and are eager to see solutions. Researchers who exhibit leadership in promising directions will be rewarded in many ways (including perks such as opportunities to give keynote talks).

Senior researchers also have a responsibility to set directions for younger researchers. Up-to-date introductory readings are scarce. Introductory courses are in a state of flux. New editions of popular text books will expand coverage of modern methods, likely at the expense of traditional topics. Discussions of co-reference these days are more likely to mention BERT than disjoint reference (Kiparsky, 2015) and c-command (Reinhart, 1981). That said, it is important to give the next generation as broad a perspective as possible since the future is likely to be quite different from the present.

### 4.4 Paths Forward for Younger Researchers

We have more to say for younger researchers because there are more differences of opinion. One of the reviewers argued that younger researchers should focus on smaller venues (workshops), and avoid the temptation to play it safe. While there is much to be said for that position, we suspect that many younger researchers will choose to play it safe, and therefore it may be useful to provide advice for those that choose that path, as well as those that choose alternative paths.

Researchers towards the start of their careers face a special set of problems. The easiest way to establish credentials is to make incremental changes in currently-fashionable methods, for which the crucial thing is mastery of the relevant techniques. This has always been difficult, and the alchemical complexity of modern deep learning technology makes it especially difficult today.

Contributing to a more innovative project sends a more powerful message, but this is more of a gamble, since such projects typically take more time and often fail, or at least fail to win acceptance. So younger researchers need to keep an eye open for promising new developments, while demonstrating their ability to work within the currently standard framework.

The publish or perish rat race is more intense than ever before. Conferences are big and growing. Submissions are way up. Senior researchers are encouraging people to spend more time on each paper (and publish less). Younger researchers believe (perhaps correctly) that they cannot afford to do that. The old cliche, publish or perish, needs to be updated to publish every month or perish. This rat race is not good for anyone, especially younger researchers who are caught in the middle of the maze.

Given these realities, it is probably safer to set relatively modest goals. Go for singles rather than home runs. It can be tempting to swing for the fences, but lead off batters tend to have better batting averages than home run sluggers. It is probably wise, when starting out, to spend more time on smaller conference-size projects that are more likely to work out, and avoid taking chances on great questions such as:

1. compositionality,
2. causality (Pearl, 2009),
3. long distance dependencies (Chomsky, 1957)

Questions that have been open for too long are unlikely to be solved quickly. Given that young researchers need to make progress quickly (or perish), it might be wise to focus of low hanging fruit, and leave great questions for more establish researchers that can afford to take more chances on topics that may take a while to produce results (if at all).

That said, it is possible that recent progress in deep nets (LeCun et al., 2015) might make it possible to make progress in the near term on some great questions that have been open for a long time. Much of the appeal of vector representations of meaning (such as Word2Vec, BERT, ELMO, etc.) involves compositionality. Some of these papers even mention the word compositionality in the title: Mikolov et al. (2013c). Datasets such as SCAN^71^ (Lake and Baroni, 2017) make it easier to write papers on compositionality.

Causality is mentioned in the title of a recent paper (Bengio et al., 2019) that suggests a connection between gradients and causality. Machine learning models tell us how to make predictions based on our current understanding of the world, but gradients tell us how to change that understanding. Given that the world is rarely the way that we currently understand it to be, gradients and causality could play an important role in closing the gap between our beliefs and reality.

Transformer models (Jaderberg et al., 2015) and attention (Vaswani et al., 2017) offer a promising new perspective on long distance dependencies. It is now possible to capture dependencies over much larger windows than traditional ngram methods that Chomsky (1957) was arguing against. BERT uses 512 word windows, considerably larger than traditional trigram windows.

We came of age in a more relaxed time when people felt they could afford to think big thoughts. But it was also a time of rebellion. It was natural in those days, especially given strong personalities like Chomsky, to see ideas as clashing, and to reject our teachers, just as they had rejected their teachers. It was popular to dismiss the establishment with cliches like “don’t trust anyone over 30.” These days, that cliche has been replaced with a new one: “ok boomer.” The sentiment may be similar, but the tone is completely different: more of an eye roll than violent rebellion.

Either way, curiosity is a natural part of growing up. Kids question everything, especially the smartest kids. When they ask, why do we do it that way, they hate lame answers like: “that’s the way it has always been done.” Rejection is a natural part of growing up. Maybe it is just a phase that we all live through. Whether it is a productive phase or not depends on what we do with it. Rejecting lame answers can lead to deep insights, but rejections can also be lame excuses for laziness. Why do we need to study so much, and learn traditional methods, if we know we are going to reject them? It is rarely a good thing to shut down, and exclude potentially productive paths forward. Traditional methods (that have stood up to the test of time) are more likely to solve your problem than whatever was posted on the archives last night.

As mentioned above, deep nets can be viewed as a rejection of the establishment, which can be viewed positively or negatively. On the positive side, end-to-end systems can be viewed as a way to make progress on challenging tasks, finessing around potentially distracting details that have proved challenging for traditional methods. But on the other hand, end-to-end systems can be viewed as a lame excuse for ignorance (about traditional topics in speech science, linguistics, etc.), and to skip various (tedious) steps in traditional best practices such as documentation and unit testing. Often the intermediate representations are present in so-called end-to-end systems, but they aren’t that well understood or documented, and consequently, they aren’t very well covered by unit testing, leading to fragile systems that are difficult to debug.

Research requires just the right combination of curiosity and focus. End-to-end systems make it easy to focus on some matters (end-to-end performance on a particular task), but leave relatively little room for curiosity. Why does the system work as well as it does? Intermediate representations make it easy to factor large tasks into more manageable sub-tasks, each of which can be studied (and evaluated/tested) independently. There is considerable merit to both positions. System testing, for example, is more credible than unit testing, if we want to measure end-to-end performance, but unit testing tends be useful for setting priorities. Do we want to invest more in this component or that component? More generally, a better together inclusive ensemble of perspectives is likely to be more productive than extreme positions that reject one position or another.

Kuhn (2012) ^72^ is massively cited (more than 100k citations in Google Scholar) because his observations offer helpful constructive advice to younger researchers starting out in many fields. The 2012 version appeared on the 50th anniversary of the first edition. Kuhn studies the evolution of Physics over time. People once believed that the earth was the center of everything. It took some time for the field to appreciate that the earth is not even the center of the solar system, let alone the Universe. The process involved a lengthy progression of paradigm shifts, with some common themes that many younger researchers find helpful. In particular, the early adopters of paradigm shifts tend to be younger researchers with relatively little invested in the status quo. Senior researchers, on the other hand, tend to resist change because they are more heavily invested in precedent.

Kuhn suggested that a successful paradigm shift needs to satisfy two criteria. The first seems obvious and hardly worth mentioning: a successful paradigm shift needs to demonstrate some initial promising results early on. But the second criterion is non-obvious and perhaps counter-intuitive: a successful paradigm shift needs to leave room for students to contribute and benefit by doing so.

It may be a bit ironic that the rejection of the past ought to be as inclusive as possible of future leaders of the field (today’s students and younger researchers). But it makes sense. Students that invest early in the next new paradigm will do well. Like the stock market, early investors in the next new thing do well. Papers are like stocks. Papers that adopt the next new paradigm are cited more than papers that invest later. By construction, the last paper on a topic is not cited much.

It has been our experience that it can be good for one’s career to get into an area just before it is about to take off. As mentioned above, there were few empirical papers in 1990, and few non-empirical papers in 2000. This timing worked out well for us. We started publishing empirical papers in 1988, and created the Linguistic Data Consortium (LDC) in 1992.

Kuhn’s advice played an important role when we were reviving empiricism in the 1990s. These days, EMNLP has evolved into a major conference, but it was far from obvious at the time that EMNLP would become as important as it has. When we first created EMNLP, the E-word (empiricism) was extremely controversal. Empricism was largely a rejection of the establishment (the rationalism of our teachers). We knew we were onto something when younger researchers were more receptive to what we were saying than senior researchers. We realized, even then, that the next generation of younger researchers would have more influence over the future of the field than the last generation of senior researchers.

What does this mean more specifically in CL today? To address this question, we thought it would be useful to call out a few specific promising paths forward, where there are already quite a number of publications and there will likely be many more in the next few years:

1. Universal Dependencies (UD) (Nivre et al., 2016): According to the web site,^73^ UD is a framework for consistent annotation of grammar (parts of speech, morphological features, and syntactic dependencies) across different human languages. There is an emphasis on inclusiveness, with over 200 contributors producing more than 100 treebanks in over 70 languages.
2. Contextual Embeddings: Embeddings such as BERT (Devlin et al., 2018) and ERNIE (Sun et al., 2020) are topping leader boards on tasks such as GLUE (see footnote 16). Contextural embeddings are believed to capture context more effectively than static embeddings such as Word2Vec (Mikolov et al., 2013a; Mikolov et al. 2013b; Mikolov et al. 2013d) and GloVe (Pennington et al., 2014). Recent work such as Clark et al. (2019) suggests different parts of contextual embeddings are capturing different linguistic aspects of context.
  a. Theory: More generally, what are nets doing? And why are they as effective as they are? Much has been written on this topic. Information Bottleneck (Tishby et al., 2000) is a particularly attractive way forward. See Tegmark and Wu (2020) for a recent study that finds new value in old ideas.
  b. Practice: We know from work in the 1970s and 1980s that “understanding” can be engineered for limited conceptual and contextual domains—at a cost (Chandioux, 1976; Chandioux, 1989). Attempts to scale up semantic grammars have been largely unsuccessful, though there are clear connections between Apple’s SIRI and earlier work on semantic grammars at SRI (Hendrix et al., 1978).^74^. There may be opportunities to revisit limited domains in areas such as EHR (electronic health records), given recent advances such as BERT, and especially BioBERT (Lee et al., 2020).

## 5 Conclusions

The intellectual climate is in a state of flux. It is the best of times and the worst of times. There are many successes. Conferences are bigger than ever before (perhaps too big). Fortune 500 companies are taking AI very seriously. Governments and industry around the world are investing big time in what we do.

That said, we are still far away from accomplishing DARPA’s 1962 mission statement, especially the last line on robustness. DARPA and others are betting that the time is ripe for change. We do not need more “cat detectors.” There are many promising paths forward for all parties including: funding agencies, managers in industrial research, senior researchers and especially younger researchers. The future depends largely on younger researchers who will soon be the leaders of the field.

## Footnotes

1
https://scholar.google.com/citations?view_op=top_venues&hl=en&vq=eng_computationallinguistics

2
https://www.aclweb.org/portal/what-is-cl

3
https://www.aclweb.org/anthology/

4
https://quoteinvestigator.com/2013/10/20/no-predict/

5
https://github.com/openai/gpt-3

6
https://en.wikipedia.org/wiki/Eddington_experiment

7
https://www.eye-on.ai/podcast-017

8
https://youtu.be/gG5NCkMerHU?t=355

9
https://en.wikipedia.org/wiki/Artificial_intelligence

10
https://plato.stanford.edu/entries/associationist-thought/

11
https://www.youtube.com/watch?v=gG5NCkMerHU&t=3240

12
https://youtu.be/RYsTv-ap3XQ?t=775

13
https://en.wikiquote.org/wiki/Fred_Jelinek and http://www.lrec-conf.org/lrec2004/doc/jelinek.pdf

14
https://www.eye-on.ai/podcast-017

15
https://paperswithcode.com/area/natural-language-processing

16
https://super.gluebenchmark.com/

17
https://explainml-tutorial.github.io/

18
https://github.com/Eric-Wallace/interpretability-tutorial-emnlp2020 and https://www.youtube.com/watch?v=gprIzglUW1s

19
https://wordnet.princeton.edu/

20
https://www.nltk.org/howto/wordnet.html

21
https://babelnet.org/

22
https://wordnet.princeton.edu/

23
https://sigchi.org/community-event/a-future-of-work-for-the-invisible-workers-in-a-i/ and https://www.youtube.com/watch?v=9K-s_eFt4ag

24
https://github.com/Sujit-O/pykg2vec

25
https://github.com/google-research/bert/blob/master/multilingual.md\#list-of-languages and https://huggingface.co/transformers/pretrained_models.html

26
https://medium.com/syncedreview/baidus-ernie-tops-google-s-bert-in-chinese-nlp-tasks-d6a42b49223d

27
https://awards.acm.org/about/2018-turing

28
https://en.wikipedia.org/wiki/History_of_Google

29
https://www.scmp.com/tech/china-tech/article/2154437/true-dominance-chinas-baidu-alibaba-and-tencent-revealed-and-how

30
https://en.wikipedia.org/wiki/Smartphone

31
https://en.wikipedia.org/wiki/Siri

32
https://voicebot.ai/2018/03/28/timeline-voice-assistant-smart-speaker-technology-1961-today/

33
https://aws.amazon.com/

34
https://www.ldc.upenn.edu/about

35
http://www.elra.info/en/about/

36
https://www.youtube.com/watch?v=x7psGHgatGM, http://www.argmin.net/2017/12/05/kitchen-sinks/ and http://www.argmin.net/2017/12/11/alchemy-addendum/

37
https://www.rand.org/pubs/papers/P3244.html

38
https://syncedreview.com/2017/12/12/lecun-vs-rahimi-has-machine-learning-become-alchemy/

39
https://www.youtube.com/watch?v=93Xv8vJ2acI?t=720

40
https://youtu.be/0tEhw5t6rhc?t=1079

41
https://en.wikipedia.org/wiki/Weapons_of_Math_Destruction

42
https://www.cambridge.org/core/journals/natural-language-engineering/article/emerging-trends-i-did-it-i-did-it-i-did-it-but/E04A550C6DFF0154C684888B7B9F68EA

43
https://www.ias.edu/events/deep-learning-2019

44
https://www.youtube.com/watch?v=gG5NCkMerHU

45
https://www.youtube.com/watch?v=cWzi38-vDbE?t=1200

46
https://www.eye-on.ai/podcast-017

47
https://engineering.nyu.edu/news/revolution-will-not-be-supervised-promises-facebooks-yann-lecun-kickoff-ai-seminar

48
https://en.wikipedia.org/wiki/Information_Processing_Techniques_Office

49
https://events.sa-meetings.com/ehome/377644/804518/

50
https://www.youtube.com/playlist?list=PL6wMum5UsYvb-6h-KQi8YYb9UQY8SzNKi

51
https://venturebeat.com/2018/10/08/darpas-betting-2b-on-your-next-ai-innovation/

52
https://www.darpa.mil/work-with-us/ai-next-campaign

53
https://www.nsf.gov/about/history/nsf50/science_policy.jsp

54
https://epsrc.ukri.org/research/ourportfolio/researchareas/natlangproc/ and https://www.healthcareitnews.com/news/emea/uk-government-give-20m-funding-boost-ai-research

55
http://www.snf.ch/en/researchinFocus/newsroom/Pages/news-191022-research-on-artificial-intelligence.aspx

56
https://ec.europa.eu/digital-single-market/en/news/towards-vibrant-european-network-ai-excellence

57
https://ec.europa.eu/digital-single-market/en/news/artificial-intelligence-79-partners-21-countries-develop-ai-demand-platform-eu20-million-eu

58
https://www.technologyreview.com/s/609038/chinas-ai-awakening/

59
https://ec.europa.eu/info/sites/info/files/commission-white-paper-artificial-intelligence-feb2020_en.pdf

60
https://www.mckinsey.com/featured-insights/europe/ten-imperatives-for-europe-in-the-age-of-ai-and-automation

61
https://www.csis.org/analysis/chinas-emerging-data-privacy-system-and-gdpr

62
https://acl2020.org/blog/general-conference-statistics/

63 As of ACL-2020, Britain was still part of the European Union. In fact, there were slightly more than 600 submissions from the European Union (including Britain). If we exclude Britain, then there were almost 450 submissions from the rest of the European Union. Britain was the largest country (by ACL-2020 submissions) in Europe, with about 25% of the “European” submissions.

64
https://www.scmp.com/news/china/science/article/2165004/european-artificial-intelligence-experts-unite-bid-challenge-us

65
https://ec.europa.eu/jrc/en/publication/artificial-intelligence-european-perspective

66
https://github.com/google/TextNormalizationCoveringGrammars

67
https://www.youtube.com/watch?v=pA5qFJyeqDo

68
https://www.youtube.com/watch?v=usRf7fYDgmw&feature=youtu.be&t=360

69
https://github.com/brendenlake/omniglot

70
https://en.wikipedia.org/wiki/Generative_adversarial_network

71
https://github.com/brendenlake/SCAN

72
https://www.nemenmanlab.org/∼ilya/images/c/c5/Kuhn-1970.pdf

73
https://universaldependencies.org/

74 See https://www.sri.com/hoi/siri/ for more on the connection between SIRI and SRI. See http://archive.computerhistory.org/resources/text/Oral_History/Hendrix_Gary/Hendrix_Gary.oral_history.2004.102657945.pdf for historical connections between Q&A, SRI and Semantic. Semantic was originally a natural language company. Q&A was successful enough that the company survived long enough to find a more successful business.
